# Prospective associations between loneliness and disordered eating from early adolescence to adulthood

**DOI:** 10.1002/eat.23793

**Published:** 2022-08-11

**Authors:** Laura Cortés‐García, Rubén Rodríguez‐Cano, Tilmann von Soest

**Affiliations:** ^1^ PROMENTA Research Center, Department of Psychology University of Oslo Oslo Norway; ^2^ Norwegian Social Research Oslo Metropolitan University Oslo Norway

**Keywords:** adolescence, bidirectional association, disordered eating, gender, loneliness, random intercept cross‐lagged panel model, young adulthood

## Abstract

**Objective:**

Despite findings from numerous cross‐sectional studies suggesting a substantial association between loneliness and different types of disordered eating, much remains unknown about the impact of confounding, the order of cause and effect, and gender differences in the relationship. Thus, this study followed a large, population‐based, mixed‐gender sample through adolescence and young adulthood, applying a random intercept cross‐lagged panel model (RI‐CLPM) approach to examine the bidirectional prospective associations between loneliness and disordered eating while ruling out the effect of unmeasured time‐invariant confounders.

**Method:**

A Norwegian sample of *N* = 2933 adolescents (54.2% female) was examined across four time points (T1, *M*_age_ = 15.44, grades 7–12; T2, *M*_age_ = 16.93; T3, *M*_age_ = 21.84; and T4, *M*_age_ = 28.33) from 1992 to 2005 using RI‐CLPMs for overall disordered eating and specific forms for disordered eating (dieting and bulimic symptoms). Multigroup structural equation models were used to assess gender differences.

**Results:**

For male participants, high levels of loneliness at T1 predicted more overall disordered eating and more dieting at T2. Meanwhile, among female participants, disordered eating and bulimic symptoms at T2 predicted more loneliness at T3, whereas loneliness at T3 predicted more disordered eating and bulimic symptoms at T4, and vice versa.

**Discussion:**

The findings suggest a pattern of bidirectional associations between loneliness and disordered eating that varies by time points, gender, and type of eating problem. Preventive interventions and treatment should consider social factors involved in the onset and maintenance of eating problems in male adolescents and young adult women.

**Public Significance:**

This study contributes to the existing knowledge by examining for the first time the dynamic nature of the association between loneliness and disordered eating while accounting for all time‐invariant confounding. Our findings reveal a pattern of bidirectional associations between loneliness and disordered eating that appears to vary by developmental period, gender, and type of eating problem. Our findings suggest that social factors have to be taken into account when designing prevention strategies aimed at disordered eating.

## 1 INTRODUCTION

Disordered eating or subsyndromal forms of eating disorders (EDs), such as fasting, vomiting, and laxative use for weight loss are commonly experienced during adolescence and early adulthood (e.g., Keel, 2018; Slane et al., 2014), and such behavior presents significant health hazards beyond increasing the risk for later EDs (Ackard et al., 2011; Stice et al., 2009). Identifying risk factors for the emergence and maintenance of disordered eating is crucial because it can inform interventions to reduce disordered eating and its consequences.

In this regard, increasing attention has fastened upon loneliness (i.e., feeling socially disconnected from others; Cacioppo & Patrick, 2008) as a potential source of mental health problems, including disordered eating (Beutel et al., 2017; Levine, 2012). However, even though research has revealed a robust association between loneliness and disordered eating (e.g., Levine, 2012), we know little about the longitudinal nature of this association. This study aimed to provide novel knowledge on this issue by means of state‐of‐the‐art longitudinal modeling across adolescence and young adulthood. We cover these developmental periods because adolescence and young adulthood represent not only a crucial stage for the onset of disordered eating (Keel, 2018; Slane et al., 2014) but also show elevated levels of loneliness (von Soest et al., 2020).

To date, cross‐sectional studies have supported an association between loneliness and different forms of disordered eating. For instance, Rotenberg and Flood (1999) reported that feelings of loneliness were associated with dietary restraint as well as higher food consumption. Likewise, scholars have found perceived loneliness to be related to emotional eating (Raspopow et al., 2013)—which is a precursor of binge eating (Stice et al., 2000)—and excessive calorie consumption (Henriksen et al., 2014; Mason, 2020). In a study focusing on college students, Wright and Pritchard (2009) showed that loneliness was positively related to disordered eating. In the same vein, perceived social problems and low levels of attachment with peers have been linked to more disordered eating in adolescents and young adults (Pritchard & Yalch, 2009; Schutz & Paxton, 2007).

The association between loneliness and disordered eating may be understood in several ways. It has been suggested that loneliness may be a source of disordered eating. For example, in accordance with the Interpersonal Model of EDs (Burke et al., 2018; Wilfley et al., 2000), individuals experiencing loneliness may resort to bulimic behavior and binge eating as a way to distract themselves from negative affect while also attempting to fill the emotional and social needs they may be encountering (Grant, 2008; Levine, 2012). Lonely people may also engage in restrictive eating or dieting as a way to numb their emotions and recreate a sense of control over their lives (Cain et al., 2008; Faber et al., 2018). Conversely, disordered eating may give rise to social isolation and feelings of loneliness (Levine, 2012; Treasure et al., 2011). Specifically, individuals with disordered eating may tend to avoid contact with peers and family in order to hide unhealthy eating behaviors (Levine, 2012). For instance, research has shown that bulimic symptoms are often associated with intense feelings of shame and guilt (Stice et al., 2000), which may lead individuals who engage in binge eating and purging to socially withdraw themselves (Basile, 2004; Rotenberg et al., 2013). Moreover, individuals who engage in restrictive dieting may avoid social gatherings since socializing often revolves around the consumption of food (Levine, 2012; Woolley et al., 2020). Lastly, the association between loneliness and disordered eating may be explained by the effects of potential time‐invariant confounders, such as common genetics (Day et al., 2018; Wade & Bulik, 2018), personality and temperamental factors (Buecker et al., 2020; Cassin & von Ranson, 2005), and response‐style biases (Decaluwé & Braet, 2004). Therefore, the association between loneliness and disordered eating may conceivably diminish once such potentially confounding factors are accounted for.

Accordingly, longitudinal studies designed to disentangle the temporal dynamics of the associations are needed to provide information about the nature of the loneliness–disordered eating association. However, to the best of our knowledge, only two studies to date have examined the prospective association between loneliness and disordered eating. Through participants' self‐report daily diaries, Mason et al. (2016) found that perceived social isolation was predictive of greater binge eating among college women who completed measures nightly each day for 14 days. Similarly, in a study that featured a large population‐based sample of youth, Abebe et al. (2014) identified loneliness as a prospective risk factor for disordered eating during adolescence and young adulthood. However, neither study examined reverse temporal associations whereby disordered eating may function as risk factor of loneliness. Also, the two studies did not rule out important potential time‐invariant confounders.

The hypothesized effects of loneliness on disordered eating or vice versa are stipulated to take place at the within‐person level, whereby a person's feeling of loneliness at a given time is expected to predict changes in that same person's disordered eating (or vice versa). However, until recently, cross‐lagged panel models (CLPMs) have been primarily used to disentangle potential reciprocal longitudinal associations between psychopathology and potential risk factors (Selig & Little, 2012), even though they do not separate between‐person variation from within‐person variation in longitudinal data (Hamaker et al., 2015). As a result, parameter estimates derived from traditional CLPM approaches are difficult to interpret meaningfully when examining individual processes across time (Berry & Willoughby, 2017). We are the first to address this limitation by applying the recently developed random intercept CLPM (RI‐CLPM) to effectively disaggregate longitudinal data into its between‐ and within‐person sources of variance (Hamaker et al., 2015; Mulder & Hamaker, 2020) when examining the association between loneliness and disordered eating. This modeling framework allows the estimation of cross‐lagged effects that are not confounded by any potential time‐invariant confounders.

A further limitation of the existing research is the scarcity of studies examining both male and female samples. In one such study, Rotenberg and Flood (1999) argued that, particularly in women, loneliness might be one of the motives responsible for dietary restraint. Similarly, Masheb and Grilo (2006) reported that, among patients with binge‐ED, women were more likely than men to report overeating in response to loneliness. In contrast, Pritchard and Yalch (2009) found that loneliness was related to disordered eating in men but not in women. In the same vein, Abebe et al. (2014) found that loneliness was a male‐specific predictor of disordered eating. Thus, more research is necessary to better elucidate whether the association between loneliness and disordered eating differs across genders during the transition from adolescence to young adulthood.

The present study aimed to examine for the first time the bidirectional associations between loneliness and different forms of disordered eating from adolescence to young adulthood while ruling out all time‐invariant confounding by means of state‐of‐the‐art longitudinal modeling techniques. We also tested whether such longitudinal associations and their timing would differ by gender. Generating such knowledge is a fundamental step in designing prevention strategies for both problems.

## 2 METHOD

### 2.1 Participants and procedure

Data for the present research come from the Young in Norway Study conducted in 1992 (T1; early‐middle adolescence), 1994 (T2; late adolescence), 1999 (T3; emerging adulthood), and 2005 (T4; adulthood). The initial sample at the first data collection included 12,655 students in grades 7 through 12 (*M*_age_ = 15.44, SD = 1.66). Participants were recruited from 67 representative junior and senior high schools in the country, with a response rate of 97%. At the first follow‐up 2 years later (late adolescence, *M*_age_ = 16.93, SD = 1.75), approximately half of the students had completed the 3‐year track at the junior or senior high school and had left the school they had been attending at the first data collection. Only students who had completed the questionnaires at school at the second data collection (*n* = 3844) were followed up in emerging adulthood (*M*_age_ = 21.84, SD = 1.76) and adulthood (*M*_age_ = 28.33, SD = 1.73). The response rates among students eligible for participation were 92%, 84%, and 82% in late adolescence, emerging adulthood, and adulthood, respectively. The overall participation rate, based on all eligible students at T1 who still were at their original school at T2, was 68% at T3 and 67% at T4. The study was approved by the Norwegian Regional Committee for Medical Research Ethics (reference S‐05030). All participants consented prior to study participation.

In this study, after excluding participants over 18 years of age at the first data collection, we used data from 2933 students (54.2% female) who participated at least at 1 time point in emerging adulthood and adulthood. At the first data collection, the parents' educational level (measured as the highest educational level attained by either parent) was as follows: 20.5% junior high school or less, 36.9% senior high school, 22.1% college education (not more than 3 years) and 20.5% university education. Moreover, 12.6% of the respondents reported parental occupational level (based on the highest occupational attainment by either parent) to be higher administrative position/leaders, 40.8% professional higher level, 13.0% professional lower level, 6.4% farmer/fisherman, and 27.3% manual workers. Of all respondents, 1.8% had both parents with immigration background (i.e., both parents were not born in Norway). Most of them were born in countries in Asia (56.6%), followed by those born in Western countries (22.6%), in South America (15.1%), and in Africa (5.6%).

Multiple logistic regression analyses showed that being female increased the likelihood of participating in the study in late adolescence (OR = 1.58, 95% CI [1.43, 1.75]), emerging adulthood (OR = 1.41, 95% CI [1.28, 1.56]), and adulthood (OR = 1.41, 95% CI [1.28, 1.56]). In addition, being younger significantly predicted participating in late adolescence (OR = .85, 95% CI [.83, .87]), emerging adulthood (OR = .73, 95% CI [.71, .76]), and adulthood (OR = .74, 95% CI [.73, .77]). Regarding disordered eating, lower scores in early‐middle adolescence predicted participating in the study during late adolescence (OR = .80, 95% CI [.71, .92]). Finally, lower levels in loneliness in early‐middle adolescence increased the likelihood of participating in emerging adulthood (OR = .88, 95% CI [.81, .96]) and adulthood (OR = .87, 95% CI [.80, .95]).

### 2.2 Measures

#### 2.2.1 Disordered eating

Disordered eating was measured using the 12‐item version of the Eating Attitude Test‐26 (EAT‐26; Garner et al., 1982), developed by Lavik et al. (1991). A 4‐point scale ranging from *never* (1) to *always* (4) was used. Mean scores were calculated, with high scores reflecting high levels of disordered eating. The EAT‐12 yields three factors: dieting, bulimia‐food preoccupation, and oral control. Each factor comprises four items. α coefficients for the whole scale ranged from .71 to .75 across data collection waves. Moreover, α coefficients ranged from .80 to .84 for the dieting subscale, and from .61 to .76 for the bulimia‐food preoccupation subscale. The somewhat large variation in internal consistency for the bulimia‐food preoccupation subscale was due to lower α coefficients at the earlier waves. The oral control subscale has been shown to be psychometrically weak (Fekken et al., 1988; Garner et al., 1982), and results based on this subscale are therefore not presented. The construct validity of the overall EAT‐12 has been supported in validation studies (Wichstrøm, 1995).

#### 2.2.2 Loneliness

Feelings of loneliness were assessed using a 4‐item short version of the UCLA Loneliness Scale developed by Russell et al. (1980). The short version of this scale has been reported to be reliable and to correlate highly with the full version (Russell et al., 1980). In addition, a fifth item, “I feel lonely,” was added to measure loneliness directly. This measure has been shown to have good face and predictive validity (von Soest et al., 2020). Response options ranged from 1 = *never* to 4 = *often*. This study's 5‐item scale has been used previously (e.g., Abebe et al., 2014) and α coefficients ranged from .65 to .78 in the present study, with lower coefficients at earlier data collection waves.

### 2.3 Statistical analysis

**FIGURE 1:**
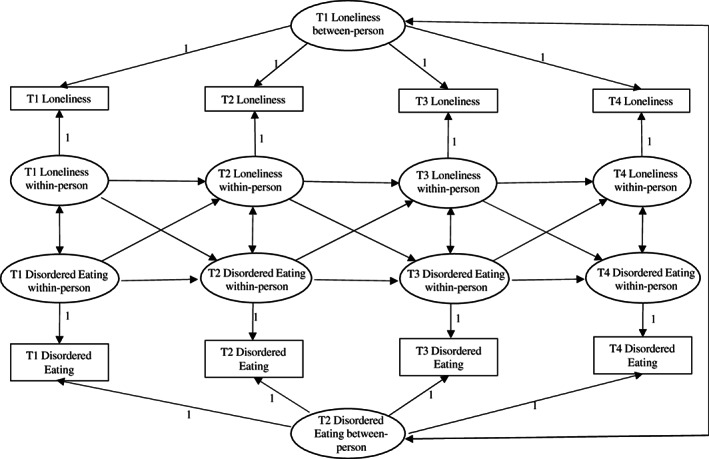
Four waves random intercept cross‐lagged panel model disentangling between‐person and within‐person effects of loneliness and disordered eating

Statistical analyses were performed using Mplus (Version 8.5). RI‐CLPM models that account for time‐invariant stability by the inclusion of random intercepts (Hamaker et al., 2015; Mulder & Hamaker, 2020) were used to test whether loneliness predicted changes in disordered eating over time and, conversely, whether disordered eating predicted loneliness. For this purpose, autoregressive, CLPMs were estimated in the structural equation modeling framework, including loneliness and disordered eating at all four time points. In addition, random intercepts for both loneliness and disordered eating were included in the models; see Figure 1 for a graphical display of the model. We used the Satorra–Bentler χ^2^‐difference test (Satorra, 2000) to assess gender differences by means of multiple group analyses. A robust maximum‐likelihood estimator was applied, and full information maximum likelihood was used to handle missing data.

## 3 RESULTS

### 3.1 Descriptive statistics

Table 1 shows that female participants had higher scores for disordered eating and loneliness than their male counterparts across all time points. In addition, loneliness was positively correlated to disordered eating (total score and both subscales) at all time points for both women and men.

**TABLE 1:** Descriptive statistics and bivariate correlations for men and women from early‐middle adolescence (T1) to adulthood (T4)

Variables	Men (*n* = 1343)	Women (*n* = 1590)	1	2	3	4	5	6	7	8	9	10	11	12	13	14	15	16
	Mean (SD)	Mean (SD)																
1. Loneliness T1	1.83 (.53)	1.90 (.55)		.53	.32	.31	.27	.25	.15	.15	.15	.14	.06	.04	.22	.19	.15	.14
2. Loneliness T2	1.79 (.56)	1.86 (.57)	.59		.44	.41	.19	.28	.20	.18	.12	.14	.10	.07	.16	.22	.19	.17
3. Loneliness T3	1.78 (.53)	1.83 (.48)	.41	.50		.55	.16	.16	.28	.18	.04	.07	.13	.05	.11	.12	.21	.14
4. Loneliness T4	1.76 (.51)	1.79 (.49)	.37	.44	.54		.12	.15	.22	.26	.04	.04	.08	.08	.09	.10	.19	.25
5. Disordered Eating T1	1.49 (.33)	1.71 (.38)	.18	.12	.13	.11		.53	.32	.29	.72	.44	.27	.20	.74	.36	.20	.24
6. Disordered Eating T2	1.43 (.31)	1.74 (.39)	.09	.12	.14	.08	.59		.39	.32	.42	.72	.34	.24	.42	.74	.26	.24
7. Disordered Eating T3	1.44 (.29)	1.71 (.39)	.07	.10	.20	.15	.34	.44		.53	.29	.35	.75	.44	.19	.26	.66	.38
8. Disordered Eating T4	1.48 (.35)	1.62 (.39)	.10	.15	.18	.26	.28	.32	.50		.27	.31	.46	.80	.21	.22	.38	.74
9. Dieting T1	1.52 (.56)	2.02 (.71)	.08	.05	.07	.05	.78	.55	.33	.28		.62	.41	.32	.33	.20	.15	.19
1. Dieting T2	1.44 (.52)	2.08 (.72)	.04	.05	.06	.02	.50	.80	.38	.29	.69		.50	.37	.25	.34	.17	.20
11. Dieting T3	1.59 (.58)	2.17 (.67)	.05	.07	.13	.08	.31	.40	.81	.44	.43	.51		.59	.14	.20	.31	.29
12. Dieting T4	1.80 (.70)	2.17 (.73)	.04	.09	.10	.15	.25	.28	.41	.84	.34	.37	.52		.13	.16	.25	.42
13. Bulimia T1	1.39 (.41)	1.49 (.47)	.16	.12	.12	.09	.75	.41	.26	.20	.42	.30	.21	.16		.47	.22	.25
14. Bulimia T2	1.35 (.40)	1.54 (.49)	.08	.12	.13	.07	.47	.77	.36	.25	.38	.48	.29	.19	.50		.30	.25
15. Bulimia T3	1.20 (.31)	1.40 (.50)	.00	.06	.18	.13	.29	.38	.82	.41	.26	.30	.56	.30	.30	.42		.43
16. Bulimia T4	1.23 (.36)	1.32 (.46)	.06	.10	.17	.23	.25	.28	.43	.78	.25	.25	.35	.52	.24	.29	.48	

*Note*: Pearson correlations for women under the diagonal. Pearson correlations for men above the diagonal. Intercorrelations of *r* = |.06| or above are statistically significantly different from zero at *p* < .001.

For loneliness, the intra‐class correlation (ICC) was .78, indicating that 78% of the variance in loneliness was due to variation between persons, whereas 22% was due to variation in loneliness within persons across time points. The ICC for disordered eating was .77 whereas examination of the subscales of the EAT‐12 yielded ICC values of .82 and .69 for dieting and bulimia and food preoccupation, respectively.

### 3.2 Prospective associations between loneliness and disordered eating

**FIGURE 2:**
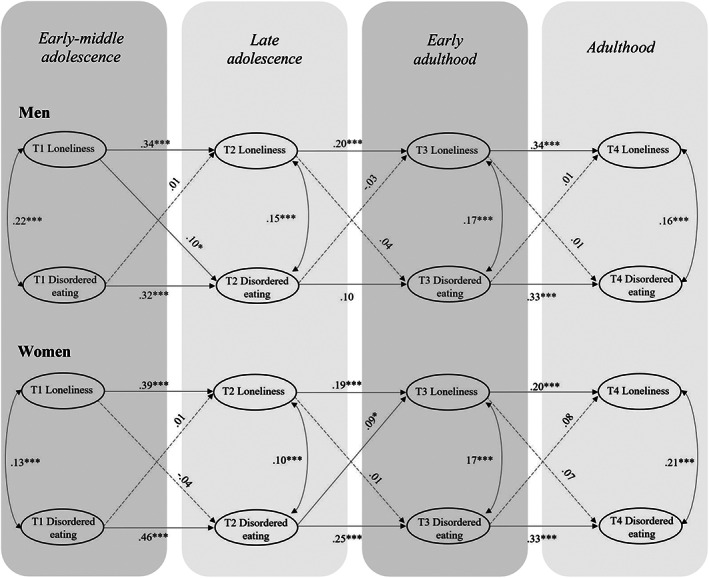
Multigroup random intercept cross‐lagged panel model (RI‐CLPM) examining the association between loneliness and disordered eating for men and women. Only within‐person effects are depicted

We used the mean scores of the EAT‐12 scale to estimate a multigroup RI‐CLPM for the associations between loneliness and disordered eating for both females and males. The model fit of the model was good (see Table 2). Results from the RI‐CLPM multigroup analyses can be found in Figure 2. At the between level, the correlation between the intercepts was significant for both males (*r* = .47, *p* < .001) and females (*r* = .27, *p* < .001), suggesting that individuals who had more disordered eating across the four measurement points reported in general more loneliness as well. At the within level, for boys, loneliness in early‐middle adolescence predicted more disordered eating in late adolescence, thereby indicating that within‐person deviations in loneliness predicted within‐person changes in disordered eating from early‐middle to late adolescence. In contrast, for girls, disordered eating in late adolescence predicted more loneliness in emerging adulthood on the within‐person level. Testing gender‐specific associations for one parameter at a time showed that the cross‐lagged association from loneliness in early‐middle adolescence to disordered eating in late adolescence differed significantly across gender (∆χ^2^ (1) = 6.82, *p* = .009), being significantly stronger in boys than in girls.

**TABLE 2:** Model fit indices of multigroup random intercept cross‐lagged panel models (RI‐CLPM) examining the longitudinal associations between loneliness and three types of eating symptomatology

	χ^2^	df	RMSEA (90% CI)	SRMR	CFI	TLI
Model 1: Disordered eating	29.23	18	.021 (.003, .034)	.018	.997	.992
Model 2: Dieting	28.26	18	.020 (.000, .033)	.016	.998	.994
Model 3: Bulimia‐food preoccupation	35.69	18	.026 (.013, .038)	.019	.995	.986

Abbreviations: 90% CI, 90% confidence interval of RMSEA; CFI, comparative fit index; df, degrees of freedom; RMSEA, root‐mean‐square error of approximation; SRMR, standardized root‐mean‐square residual; TLI, Tucker–Lewis index.

### 3.3 Prospective associations between loneliness and dieting

**FIGURE 3:**
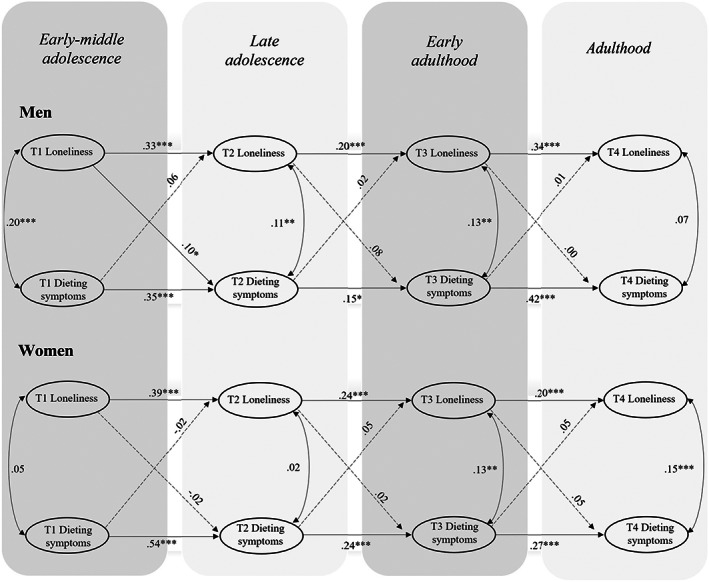
Multigroup random intercept cross‐lagged panel model (RI‐CLPM) examining the association between loneliness and dieting for men and women. Only within‐person effects are depicted

Next, we conducted RI‐CLPM analyses with the dieting subscale. The overall model fit for the two‐group model was good (see Table 2). Figure 3 displays the results from gender‐specific analysis. At the between level, no significant associations were observed between the two intercepts of loneliness and dieting. At the within the level, the analysis revealed a significant cross‐lagged association from loneliness in early‐middle adolescence to dieting symptoms in late adolescence for boys only, indicating loneliness on the within‐person level to be associated with within‐person changes in dieting during this period. The results of testing gender‐specific associations showed that the cross‐lagged associations from loneliness in early‐middle adolescence to dieting in late adolescence were significantly stronger for boys than for girls, ∆χ^2^ (1) = 4.84, *p* = .027.

### 3.4 Prospective associations between loneliness and bulimic symptoms

**FIGURE 4:**
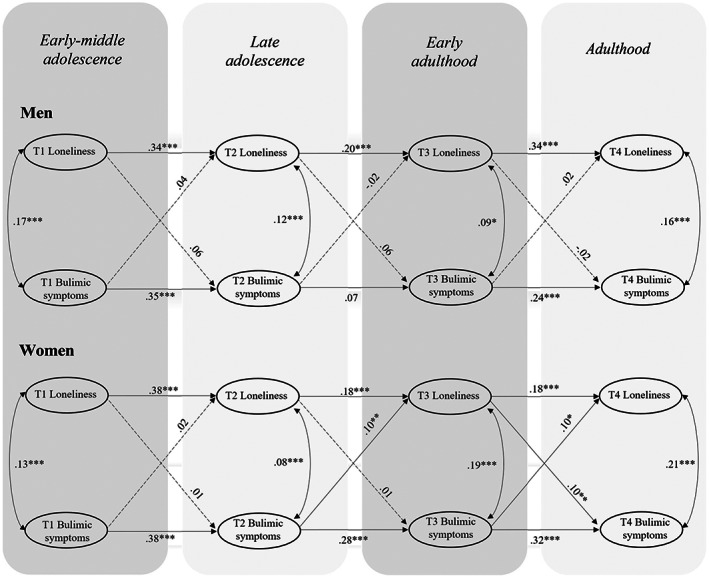
Multigroup random intercept cross‐lagged panel model (RI‐CLPM) examining the association between loneliness and bulimic symptoms for men and women. Only within‐person effects are depicted

Regarding multigroup RI‐CLPM results for loneliness and bulimic symptoms, the model fit for the two groups was also good (see Table 2). Figure 4 presents the results. At the between level, the associations between the intercepts were significant for both males (*r* = .43, *p* < .001) and females (*r* = .19, *p* = .010). At the within the level, significant cross‐lagged paths were found only among females, where bulimic symptoms in late adolescence predicted more loneliness in emerging adulthood, loneliness in emerging adulthood predicted more bulimic symptoms in adulthood, and bulimic symptoms in emerging adulthood predicted more loneliness in adulthood. Constraining one parameter at a time revealed a significantly stronger cross‐lagged association from loneliness in emerging adulthood to bulimic symptoms in adulthood for women than for men, ∆χ^2^ (1) = 5.28, *p* = .025.

## 4 DISCUSSION

Following a large population‐based sample throughout 13 years covering adolescence and young adulthood, this study used RI‐CLPM to examine, for the first time, the longitudinal, within‐person associations between loneliness and disordered eating among males and females. Within‐person, cross‐lagged estimates showed that the associations varied by gender and developmental period. Among boys, loneliness in early‐middle adolescence was prospectively related to more disordered eating and dieting 2 years later. Among women, we identified bidirectional prospective associations from early adulthood to adulthood between loneliness and bulimic symptoms. Moreover, among women, late adolescent disordered eating and bulimic symptoms were related to higher levels of loneliness in early adulthood, whereas no significant associations were observed earlier in adolescence for any type of disordered eating.

The significant within‐person associations between loneliness in early‐middle adolescence and disordered eating in late adolescence among boys indicate that feelings of loneliness may render boys vulnerable to developing disordered eating during adolescence, even when adjusting for all time‐invariant confounding. Of note, the significant association with male dieting in adolescence indicates that the relationship between loneliness and the overall disordered eating score is mainly driven by dieting behavior. Early and middle adolescence entails profound physical, cognitive, and affective developmental changes and is therefore a period characterized by heightened vulnerability to problems in regulation of emotions and behaviors, particularly among boys (Steinberg, 2005). At this age, adolescents are also more susceptible to peer influence and the need to belong to a social group becomes increasingly important (Pattiselanno et al., 2015). Feelings of loneliness may therefore be of particular importance for guiding emotions and behavior, including eating symptomatology, in this developmental period. In addition, boys increasingly focus on their own bodies in terms of body performance and muscularity as they navigate through adolescence (Eisenberg et al., 2012; Ricciardelli, 2012). Thus, lonely boys may attempt to become more integrated within their peer group by engaging in intense physical exercise and unhealthy eating behaviors to build muscle mass (McCabe & Ricciardelli, 2003) or reduce weight (Ricciardelli, 2012; Vincent & McCabe, 2000), which may explain the within‐person longitudinal association between loneliness and dieting in adolescence.

In contrast, no significant associations between loneliness and any form of disordered eating were found among girls in early‐middle and late adolescence. A potential explanation of this finding is that the pervasive norm for female thinness (Wertheim & Paxton, 2012) may be the main driving factor for disordered eating for many adolescent girls, independent of whether they feel socially isolated or not. Moreover, some aspects of social integration may even promote disordered eating. For example, research has revealed that adolescent girls frequently use “fat talk” when socializing (Shannon & Mills, 2015; Sharpe et al., 2013). Such negative remarks about one's own or another's appearance may, in turn, lead to more disordered eating symptoms, counterbalancing the potential negative effects of loneliness. Research has also shown that, during early‐mid adolescence, boys and girls differ in terms of stage of pubertal maturation (Tanner, 1971), whereby girls, in general, go through puberty earlier than boys (Hayward, 2003), and such differences may explain gender differences in the association between loneliness and disordered eating in adolescence. However, such potential explanations are speculative and further research examining gender differences is needed.

When examining female disordered eating later in life, we found that disordered eating prospectively predicted increased levels of loneliness, which supports our hypothesis that eating problems may indeed promote social withdrawal and loneliness among women (Fairburn & Harrison, 2003; Levine, 2012). Bulimic symptoms may be of particular importance for the development of loneliness, as indicated by the significant association between the bulimia‐food preoccupation subscale and loneliness among women. The results are in line with the notion that disordered eating in general, and bulimic symptoms in particular, are often accompanied by feelings of shame and guilt (Stice et al., 2000). Such feelings may, in turn, lead to social isolation and loneliness, especially in the late teens through the mid‐20s, a developmental period where new social relationships are formed and where people typically lack the daily companionship of either their family of origin or their family to be (Arnett, 2000). Women in this age group with disordered eating may, therefore, be particularly prone to isolate themselves from the normalizing influence of their peers and family, allowing their unhealthy eating behaviors, such as bulimic symptoms, to persist unchallenged (Levine, 2012).

Nevertheless, we also found reverse associations in that loneliness predicted increased bulimic symptoms among women. Again, the particular situation of young adulthood may increase lonely people's risk of developing bulimic symptoms: In this age group, many of those who experience loneliness may live alone; thus, they may not be exposed to the type of “social control” from family members that would make it difficult for them to maintain symptoms such as binging and purging (von Soest & Wichstrøm, 2006). Moreover, it is possible that women who isolate themselves and feel lonely to a larger degree than men struggle with bulimic symptoms, such as purging and binging, as a way to distract themselves and numb the negative emotions that result from feelings of failing in the interpersonal domain (Fairburn et al., 2003; Murphy et al., 2012).

Our results thus indicate that loneliness and bulimic symptoms are interconnected in a reciprocal fashion among women, where loneliness and bulimic symptoms exacerbate each other in a maladaptive cycle (Esplen et al., 2000; Levine, 2012). Consequently, our findings indicate that loneliness and bulimic symptoms may foster each other through a reciprocal process among women during young adulthood.

### 4.1 Limitations

Our results should be interpreted within the context of several limitations. Because participants were Norwegian, generalizing the present results to other countries and cultures should be approached with care. The study also relied solely on self‐reports from one source. However, as the rater did not change over time, common method bias is accounted for by adjusting for the random intercepts in the model. As another factor to consider, we studied disordered eating symptoms, but no information about ED diagnoses was included in the data. Therefore, it cannot be ruled out that prospective associations would differ if EDs were examined. Moreover, there have been critics questioning the validity of the EAT short version questioning its validity for measuring disordered eating among non‐clinical samples (Engelsen & Hagtvet, 1999) and especially among men (Murray et al., 2017; Sangha et al., 2019). Our study also shows that the internal consistency of the bulimia‐food preoccupation subscale and the loneliness measure was lower at earlier data collection waves than later ones. Even though this finding is consistent with previous research showing that individual difference instruments obtain lower reliability for younger than older participants (McFarland & Sparks, 1985), the heterogeneity in internal consistency may indicate that these measures are perceived somewhat differently at different ages.

Another limitation concerns the partial overlap of different developmental periods across the first and second data collections, where early‐middle adolescence could not precisely be separated from late adolescence, as the age of the participants partly overlapped at these two data collections. Moreover, although time‐invariant factors were accounted for by our modeling approach, the potential effect of time‐varying factors (e.g., negative life events such as the death of a family member) were not addressed. In particular, negative affect was not considered as a time‐varying confounder or mediator in the association between loneliness and disordered eating.

Some researchers have recently argued that the RI‐CLPM does not allow for a perfect distinction between within‐person and between‐person variance (Orth et al., 2021). Nevertheless, based on the current state of knowledge, RI‐CLPMs are the preferred state‐of‐the‐art models when examining within‐person developmental processes (Orth et al., 2021).

As with most longitudinal research, we observed attrition bias, as key variables in this study (i.e., loneliness, disordered eating, gender) were related to drop‐out, and therefore our findings should be interpreted with caution. However, simulation studies have shown that—even though nonrandom attrition affecting the results cannot be completely ruled out—associations between variables are only to a small degree affected by systematic attrition (Gustavson et al., 2012). Moreover, we applied a full information maximum likelihood procedure, which produces less biased estimates than complete case analysis (Enders & Bandalos, 2001). Finally, the effect sizes reported were small to moderate. However, large‐sized associations may not be expected, given the rather long time span involved and the rigorous control for all time‐invariant confounding in our analyses.

### 4.2 Implications for practice and research

Our findings underscore that the longitudinal associations of loneliness with disordered eating vary by timing and gender in the transition from adolescence to young adulthood. In line with the Interpersonal Model of EDs (Wilfley et al., 1997; Burke et al., 2018), the study highlights the importance of considering the social factors that may be involved in the onset of disordered eating when designing preventive and intervention efforts, particularly in adolescent boys and women in young adulthood. Nevertheless, further research exploring the intersection of other relevant time‐varying constructs, such as negative affect and negative life events, with loneliness and disordered eating is needed to better inform prevention and early intervention strategies.

Future studies should expand our research to different populations, such as clinical samples and participants from diverse locations and ethnic backgrounds. Furthermore, it would be interesting to examine whether the present findings hold when using different measures for disordered eating, such as clinical interviews, and other self‐report measures more suitable for boys, such as the Eating Disorder Assessment for Men (Stanford & Lemberg, 2012). It should also be acknowledged that loneliness could be a non‐specific/transdiagnostic risk factor for other mental health problems (Beutel et al., 2017) and therefore not specific to ED symptoms. We therefore encourage future studies to explore the nature of the prospective association between loneliness and other forms of psychopathology on a within‐person level. Finally, further work examining the temporal association between loneliness and disordered eating should consider shorter‐interval longitudinal monitoring across specific developmental periods in adolescence (i.e., early, middle, and late adolescence). Such studies could identify potential gender differences in associations at specific stages of maturation on these associations and thereby inform age‐specific prevention programs in adolescence.

## 5 CONCLUSION

This study contributes to the existing knowledge by examining the prospective associations between loneliness and disordered eating, which seems to be driven by dieting for boys and bulimic behavior for women while accounting for all time‐invariant confounding. In adolescence, more loneliness appeared to predict more disordered eating and dieting among boys. In contrast, in the transition to young adulthood, both disordered eating and bulimic symptoms predicted more loneliness among women. In addition, reciprocal associations between loneliness and bulimic symptoms appeared among women, indicating that both foster each other. Our findings highlight the complex interrelationship between loneliness and disordered eating and suggest that social factors have to be taken into account when designing prevention strategies aimed at disordered eating.

## AUTHOR CONTRIBUTIONS

**Laura Cortés‐García:** Conceptualization; data curation; formal analysis; methodology; visualization; writing – original draft; writing – review and editing. **Rubén Rodríguez Cano:** Data curation; formal analysis; writing – review and editing. **Tilmann von Soest:** Conceptualization; data curation; funding acquisition; methodology; writing – review and editing.

## FUNDING INFORMATION

This research was supported by two grants from the Research Council of Norway, Grant Numbers: 288083 and 301010.

## CONFLICT OF INTEREST

All authors declare that they have no conflict of interest to disclose.

## Data Availability

The data that support the findings of this study are available from the corresponding author upon reasonable request. The data are not publicly available due to privacy and ethical restrictions.
